# “It’s Not Just Technology, It’s People”: Constructing a Conceptual Model of Shared Health Informatics for Tracking in Chronic Illness Management

**DOI:** 10.2196/10830

**Published:** 2019-04-29

**Authors:** Lisa M Vizer, Jordan Eschler, Bon Mi Koo, James Ralston, Wanda Pratt, Sean Munson

**Affiliations:** 1 Division of General Medicine and Clinical Epidemiology School of Medicine University of North Carolina at Chapel Hill Chapel Hill, NC United States; 2 Northwestern University Chicago, IL United States; 3 Kaiser Permanente Washington Seattle, WA United States; 4 University of Washington Seattle, WA United States

**Keywords:** consumer health informatics, chronic illness, patient generated health data, patient reported outcomes, workflow, information seeking behavior, shared decision making

## Abstract

**Background:**

For many people, tracking health indicators is central to managing a chronic illness. However, previous informatics research has largely viewed tracking as a solitary process that lacks the characteristics essential to tracking in support of chronic illness management.

**Objective:**

To inform development of effective technologies that aid tracking of health indicators to support chronic illness management, this study aimed to construct a health informatics model that accurately describes the work and social context of that tracking work.

**Methods:**

As part of a larger project, we conducted semistructured interviews with 40 adults concerning their chronic illness management practices, including tracking and communication. We also assembled transcripts of 30 publicly available videos of 24 adults discussing tracking processes for managing their own chronic illness. We used qualitative methods to analyze interviews and video transcripts through the lens of ongoing personal and health informatics research.

**Results:**

We have described the people and work involved in tracking in support of chronic illness management and contributed a Conceptual Model of Shared Health Informatics (CoMSHI). Specifically, we identified the need for a health informatics model that (1) incorporates the ongoing nature of tracking work and (2) represents the social dimension of tracking for illness management. Our model depicts communication, information, collection, integration, reflection, and action work in the social context of the person with chronic illness, informal carers, health care providers, and community members.

**Conclusions:**

The resulting CoMSHI yields a more detailed and nuanced viewpoint of tracking in support of chronic illness management and can inform technology design to improve tracking tools to support people in more confident and capable chronic illness management.

## Introduction

### Background

Chronic illness, defined as “tend[ing] to be of long duration and are the result of a combination of genetic, physiological, environmental and behavioural factors” [1], is the leading cause of poor health, disability, and death, accounting for up to 86% of health care spending [2]. Over half of Americans have a chronic illness, with 25% having more than one [3]. To live well with a chronic illness, people must engage effective management strategies, including tracking behaviors, biometrics, and symptoms, within the constraints of their everyday lives [4,5], and they are turning to technology solutions in increasing numbers [6] (we refer to *people* rather than *patients* throughout this paper to acknowledge the whole person who has a chronic illness and not just their patient role, which may not resonate outside of the clinical setting; management activities take place, overwhelmingly, in the course of day-to-day living [7,8]).

Many health informatics tools—such as continuous glucose monitors, activity trackers (eg, Fitbit), heart rate monitors, and smartphone apps (eg, OnTrack)—promise to ease the work of tracking in support of chronic illness management. However, many people do not find these tools useful [9], and those who do use these tools often experience substantial barriers to effective use [10,11]. Despite these barriers, many people managing chronic illness must regularly track health indicators to maintain or improve health [6,12], whether using a digital or analog tool, and need better support for successful tracking practices. Understanding the processes related to the tracking work of people managing chronic illness is critical for developing health informatics tools that support confident, capable, and effective illness management.

As noted by health informatics researchers [13,14], however, current informatics models do not adequately represent the social context or the tracking tasks undertaken to manage chronic illness. A model accounting for the people and work involved in tracking can guide the design of technologies and services that better integrate into people’s lives and support their goals. To bridge this gap in representation, we developed an improved health informatics model through a qualitative analysis of 69 transcripts from 63 people describing their chronic illness management routines. The contribution of this study is a Conceptual Model of Shared Health Informatics (CoMSHI) describing tracking in support of chronic illness management. We delineate the components of the CoMSHI and relationships among those components with the goal of supporting the design of informatics tools that align with chronic illness tracking work [8,13,14].

### Related Literature

Our study builds on and extends the literature on tracking behavior in general, tracking for chronic illness management, and current personal and health informatics models.

#### Tracking

People engage in tracking activities for a wide range of purposes, from understanding finances to improving productivity to supporting artistic expression [10]. At the forefront of tracking technology and practice is the Quantified Self (QS) community [15], an enthusiastic group of trackers who describe themselves as an “international collaboration of users and makers of self-tracking tools.” QS members are active around the world with regional meetings and an annual conference. At meetings, trackers give *Show and Tell* presentations to describe their experiences.

Researchers study the practices of these and other trackers to understand their collection and sense-making strategies [10] and develop models of the tracking process [11,16] (discussed later in this section). People track using a variety of methods including automatic methods, such as bank logs, and manual methods, such as calendars [11]. Tools may be analog, such as paper journals, or digital, such as smartphone calendars [11]. After collecting data, people reflect on relationships among and patterns in data. Reflection can lead to insights about behavior or decisions to change behavior. However, barriers to successful tracking include difficulties with (1) deciding what data to collect and what tools to use, (2) using tools, (3) collating and formatting data, (4) understanding and reflecting on the data, and (5) formulating action plans based on the data [10,11].

#### Tracking for Management of Chronic Illness

Some QS members track to identify and solve health problems, although most health-related tracking is for general wellness. Similarly, much research focuses on tracking for general health and wellness (eg, [17-21]). Tracking has become an important and prevalent activity among the general public; the Pew Internet & American Life Project [6] recently reported that 69% of Americans track health data for themselves or a loved one.

However, many people managing a chronic illness must monitor their symptoms and their health through tracking. As of 2013, 19% of adults with no chronic illness tracked a health factor, whereas 40% of those with 1 chronic condition engaged in tracking, and 62% of adults with 2 or more chronic illnesses track one or more health factors [6]. This tracking can be an effective part of managing chronic illness, improving health outcomes [22], and communicating with health care providers [23]. Studies examining the lived experiences of tracking find that it is often coordinated or influenced by communication with health experts (eg, [24-26]), peers (eg, [14,20,21]), family members (eg, [27-29]), and colleagues and workplace programs (eg, [30]). Although much of the literature on tracking acknowledges that these practices happen in and are influenced by various social contexts, the models that guide design and study of tracking tools focus on individual tracking [11,16].

As more people managing chronic illness participate in health tracking, health care providers and researchers need to understand their work and social ecosystem. Moreover, although technology facilitates tracking, limited evidence supports the efficacy of specific tools to accomplish successful illness management [31]. Barriers to successful tracking for health include (1) insufficient support for collaboration with a provider [32], (2) difficulty making sense of data leading to lapses in tracking [16], and (3) difficulty remembering to track or deciding what to track [33]. Furthermore, many studies find that the apps available to support tracking for chronic illnesses are of poor quality [34-40], pointing to a lack of understanding of the needs of people managing chronic illness.

MacLeod et al [24] interviewed 12 people with a range of chronic illnesses who tracked some aspect of their health. They found that people wished to understand how their illnesses affected their lives within the context of information from health care providers. Mamykina et al [41] developed and tested MAHI—a mobile tracking and communication tool for people with diabetes. Their work revealed that, even with coaching from a diabetes educator, people newly diagnosed with diabetes struggled to develop self-efficacy and reflective thinking skills with regard to the data they captured. Our study sought to complement these studies by contributing an understanding of the processes by which trackers engage with a social dimension of tracking behavior.

#### Personal and Health Informatics Models

A total of 4 informatics models have articulated the tracking process: Li et al [11]; Epstein et al [16]; Swan [42]; and Murnane et al [43]. Li et al [11] and Epstein et al [16] focus on general personal informatics, whereas Swan [42] and Murnane et al [43] discuss informatics in the context of health.

Li et al [11] conducted an interview study of 11 self-trackers to derive a stage-based personal informatics model. Their model describes the process of general self-tracking for any purpose and is composed of *preparation*, *collection*, *integration*, *reflection*, and *action* stages. This model focuses solely on the stages through which a single person progresses in self-tracking. The authors highlight the dependencies between each stage: a mistake in preparation can cause someone to collect the wrong data, and these problems then cascade to the integration, reflection, and action stages. Whooley et al [44] expand on the integration stage of Li et al’s personal informatics model with a discussion of *why* people track and *how* they integrate their data. More recently, Mamykina et al [26] studied the process of self-discovery in a structured diabetes education program, showing how tracking can scaffold learning and reflection for diabetes management.

The stage-based personal informatics model describes an ideal process for tracking, but that process can break down when it encounters the realities of everyday life [45]. To describe general tracking in everyday life, Epstein et al [16] propose the Lived Informatics model. This model is based on 184 surveys and 22 interviews on self-tracking behaviors for physical activity, location, and finances. The authors refined Li et al’s model by dividing its original *preparation* stage into 2 stages: *deciding* and *selecting*. Introducing a cycle named *tracking and acting*, they describe an iterative progression through *collection*, *integration*, and *reflection*. Finally, this model anticipates that people will lapse in their tracking practice either temporarily or permanently.

Swan [42] proposes a model for Patient-driven Health Care that includes self-tracking among the actions in which a patient might engage. The patient is the only one engaging in work, and people other than the patient are included only peripherally. Furthermore, the evidence base for developing the model is unclear.

Murnane et al [43] examined applying Ecological Systems Theory [46] to the work of tracking in long-term management of severe mental illness [43]. The resulting model describes the influences and resources available to people, including close personal ties, indirect institutional influences, and an individual’s sociocultural context. They discuss how personal informatics tools and data form an *informatics layer* that can mediate interactions with these other services, though the model does not articulate activities in the tracking process.

Although researchers have used these models as a lens through which to study tracking related to health [14,47], they found that they lack elements important to the health context. Costa Figueirido et al [14] studied women making sense of infertility and found that the stage-based model did not adequately represent the fluidity of work or the collaboration in which women engaged. Mishra et al [47] studied people tracking while hospitalized and also observed that the stage-based model did not characterize the collaboration occurring around data. Costa Figueirido et al [14], Mishra et al [47], and Valdez and Brennan [13] all explicitly indicate a need for a model that more closely aligns to the unique needs of the health context.

This study addresses key gaps by constructing a model representing the work and people involved in tracking to support chronic illness management.

## Methods

### Datasets

We collected data and conducted interviews with people managing chronic illness in the following 3 groups: QS speakers presenting about managing chronic illness, adults managing type 2 diabetes, and mothers managing a child’s asthma. We chose publicly available QS videos to access a sizeable sample of expert trackers using innovative technology, and we chose to interview people in the community to gain the perspective of more typical trackers and technology users.

We obtained institutional review board approval from Group Health Research Institute, University of Washington, and University of North Carolina at Chapel Hill for collection, analysis, and reporting of data used for this study.

#### Quantified Self Cohort

For the QS dataset, we selected videos posted publicly on the QS blog [15] between January 2012 (blog inception) and December 2018 (end of data collection) that focused on managing a chronic illness, as defined in the Introduction. QS speakers are enthusiastic technology and tracking hobbyists who often characterize their tracking practices as innovative. They share their experiences at local and worldwide meetings to disseminate information about their routines and insights. In these videos, people described their work processes: information they tracked, how they analyzed and learned from that information, and when and how they shared information with others.

These presentations are meant to instruct other QSs, therefore giving us access to their expertise similar to an interview elicitation. Given their proficiency, we expected QS presentations to give us an overview of the cutting edge of how people build knowledge about chronic illness through tracking. As the videos are publicly accessible, we were not required to obtain consent for their use. Although presenter names are included on the QS blog, we chose to use anonymous identifiers for analysis and reporting.

#### Interview Cohorts

The interview participants were patients of primary care providers in Group Health owned and operated clinics (now Kaiser Permanente Washington), a large integrated health care delivery system in Washington State providing care to over 300,000 people. We recruited adults managing type 2 diabetes and mothers managing asthma for at least one child aged 12 years or younger. These 2 diagnoses were chosen because each requires daily health-related tasks and frequent contact with health care providers and health care systems (eg, scheduling appointments, filling prescriptions, scheduling lab tests, and asking questions outside of formal appointments). We used purposeful sampling to identify participants representative of the general population in the Northwest United States based on gender, ethnicity, technology use (with recorded use of a patient portal as a proxy), and education. All interview participants completed an informed consent process. We conducted semistructured interviews in each participant’s home inquiring about health goals, priorities for completing health tasks, and workflow in attaining those goals and tasks. The workflows articulated by participants included information on tracking and communication in support of health management. Group Health Research Institute contracted a *Health Insurance Portability and Accountability Act* (HIPAA)–approved outside agency to transcribe and redact audio recordings of interviews.

### Analysis

We coded transcripts with the ATLAS.ti (Scientific Software Development GmbH) software package using open coding. Our analysis was informed by tracking and personal informatics literature (to ensure construct validity) as well as themes we identified on initial read-throughs regarding tracking behavior.

Specifically, 3 authors (LMV, JCE, and BK) iterated through a subset of the data corpus, revising the list of open codes to refine the scope of the analysis and clearly define individual codes. When all coders agreed that the list of open codes sufficiently represented the themes related to the scope of the inquiry (ie, tracking behaviors and social dimensions of such behaviors), the coders converted the list of open codes to an axial coding scheme using affinity diagramming [48]. We then applied the axial codes to the complete data corpus. The resulting coded dataset, and the process of tracking that it described, was used to define a new conceptual model that describes the work and people involved in tracking to manage chronic illness. The following section first describes the analysis results then describes the new conceptual model.

## Results

### Datasets

Our dataset included transcripts of videos and interviews with people managing chronic illness, and the analysis guided the definition of the components of the tracking process supporting chronic illness management and construction of a model of this process. We collected data from 64 people managing a chronic illness. Data came from 24 QS speakers, 20 adults with type 2 diabetes, and 20 parents of a child with asthma. The QS cohort consists of highly proficient trackers and technology users, in contrast to the interview cohorts made up of mainstream trackers and technology users.

#### Quantified Self Cohort

From among the videos on the QS blog, we identified 30 publicly available videos meeting inclusion criteria, with a total running time of over 6 hours and 57 min. Videos were from 24 people; 4 speakers made 2 presentations each, and 1 speaker made 3 presentations. A total of 16 speakers were male (67%). One speaker acted as an informal carer (4%) for her child (ie, an unpaid provider of health-related care and support).

All speakers appeared to be of non-Hispanic white race and ethnicity and therefore do not represent the demographics of the general population. However, we judged that the videos still provide valuable insights, and the homogeneity of this sample is somewhat balanced by the diversity of the interview samples discussed in the next section. Table 1 describes the speakers’ gender, race, tracking role, employment, and diagnosed illnesses. The mean tracking interval that the speakers referenced was 3 years. The average video length was 13 min 50 seconds. QS videos are denoted with Q# identifiers in the quotes highlighted in the results.

**Table 1:** Quantified Self (QS) speaker demographics.

Demographics		n (%)^a^
**Gender**		
	Female	8 (33)
	Male	16 (67)
**Race and ethnicity**		
	White (non-Hispanic)	24 (100)
**Role**		
	Person with chronic illness	23 (96)
	Carer of person with chronic illness	1 (4)
**Employment**		
	Technology industry	8 (33)
	Other industry	4 (17)
	Academia	4 (17)
	Health care (eg, physicians and nurses)	3(13)
	Not reported	5 (21)
**Illness**		
	Diabetes	9 (38)
	Allergies (food or environmental)	4 (17)
	Parkinson disease	2 (8)
	Crohn disease	2 (8)
	Arrhythmia	1 (4)
	Chronic fatigue	1 (4)
	Chronic headaches	1 (4)
	Chronic neurological Lyme disease	1 (4)
	Heart valve disorder	1 (4)
	Panic disorder	1 (4)
	Restless leg syndrome	1 (4)

^a^Percentages are rounded to the nearest whole number.

#### Interview Cohorts

We enrolled 20 adults with type 2 diabetes and 20 mothers of children aged 12 years and older with asthma from among the patients of Group Health clinics. Table 2 describes participant demographics. Interviews ranged in length from 45 to 90 min with an average of about 60 min. For diabetes cohort participants with an informal carer, we invited them to participate in the interviews if possible. Diabetes cohort participants have D# identifiers in the quotes highlighted in the results. Asthma cohort participants are denoted by A# identifiers.

### Analysis

One author (LMV) transcribed the QS videos, and a HIPAA-approved vendor transcribed the interviews. We analyzed all transcripts using the qualitative analysis method outlined in the Methods section. We have discussed the tracking components we observed and described the model we constructed from these components.

#### Components of Tracking in Support of Chronic Illness Management

The primary themes emerging from our analysis consist of 2 parts—*actors* and *work*. The types of actors and work are summarized in Textbox 1.

We have discussed each actor and type of work, supported with examples from our analysis.

#### Actors

*Actors* are the *person with chronic illness*, *informal carers*, *health care providers*, and *community members*. These actors interact with each other and can all perform aspects of work, as described below. This definition extends beyond people included in the models of personal and health informatics from Li et al [11], Epstein et al [16], and Swan [42].

**Table 2:** Interview cohort demographics.

Demographics		Asthma	Diabetes
**Gender, n (%)**			
	Female	20 (100)	10 (50)
	Male	0 (0)	10 (50)
Age (years), mean		37.5	64.5
**Education, n (%)**			
	High school or less	4 (20)	8 (40)
	At least some college	16 (80)	12 (60)
**Race and ethnicity, n (%)**			
	Asian	0 (0)	2 (10)
	Black	6 (30)	6 (30)
	White (non-Hispanic)	10 (50)	10 (50)
	Other or no ethnicity given	4 (20)	2 (10)
	Hispanic^a^	1 (5)	1 (5)

^a^Hispanic ethnicity designation overlapped with other designations of race.

**Types of actors and work identified through transcript analysis.**

Model Components and Types

- Actors
  - Person with chronic illness, optionally including informal carers
  - Health care providers
  - Community members
- Work
  - Communication
  - Information
  - Collection
  - Integration
  - Reflection
  - Action

##### Person With Chronic Illness and Informal Carers

The *person with chronic illness* and *informal carers* are the actors most affected by the success or failure of chronic illness management and are therefore central to the tracking process. Informal carers are usually an unpaid spouse, partner, adult child, or parent. Carers often actively include the person with chronic illness in tracking work and may act as advocates or facilitators in managing chronic conditions. Carer involvement—which is crucial (eg, legally or financially) in certain situations, such as a parent advocating and caring for a child with chronic illness—is one example of the fundamentally social nature of tracking in support of chronic illness management and is not adequately described by previous informatics models. To this point, 1 carer was the mother of a child with type 1 diabetes, who told her son, “you’re a scientist along with us, and you’re making these discoveries” (Q25).

Other research describes the dynamics between the person with chronic illness and informal carers in more depth, especially with regard to patient portals [28,29].

##### Health Care Providers

*Health care providers* are skilled health professionals involved in a person’s care. Although most people mention physicians when talking about health care providers, our analysis also noted many types of nurses (eg, school nurses, nurse practitioners, and homecare nurses), physical therapists, pharmacists, nutritionists, and others. This is consistent with other literature on chronic illness care [49].

##### Community Members

*Community members* are nonhealth professionals with whom other actors interact. This definition is more inclusive than Swan’s, which included only peers. This actor includes the widest variety of people, such as intimate partners, friends, roommates, others with chronic illness, colleagues, or schoolteachers. Other literature describes the community of a person with chronic illness more in depth [13,50-53].

#### Work

The types of tracking *work* include *communication*, *information*, *collection*, *integration*, *reflection*, and *action*. These types of work are similar to the stage-based model [11] but add *communication* work (to incorporate interactions between actors) and redefine *preparation* to *information*. Perhaps most important for the mechanics of work processes, we observed that *unconstrained transitions* described the structure of tracking for chronic illness management better than discrete stages. This reflects both the continuous *and* social natures of work revealed in our datasets. As also described by Epstein et al [16] and Costa Figueirido et al [14], our analysis showed that different types of work can occur simultaneously. Furthermore, any actor can engage in any work, and actors often collaborate or hand off work. We have discussed each type of work, dependencies, and workflow.

##### Communication

*Communication* work encompasses interactions between actors. These interactions may involve illness-related information, tracked data, visualizations, or motivational support. This work is particularly important in management of chronic illness because of the number of actors and amount of work involved. Valdez et al [8] refer to this as *articulation work*.

People with chronic illness and carers regularly manage communication tasks with others, often leveraging others’ expertise. One mother who we interviewed had a friend who helped her better understand her child’s allergy triggers:

We went to a friend’s house and they had a dog, and my friend’s a doctor and she was like “you know, she’s having some kind of reaction to something, what’s going on?”

A4

Some people struggled with lack of technology support for communication. Although she faithfully uses tracking to help her manage type 2 diabetes, Quantified Self speaker Q30 wishes she could easily share her data with her physician and family:

I would like an option to share these data points with my primary care provider so that he can see that I’m doing well and feeling well and my numbers are reflecting that. Also I’d like to be able to share this with my family, especially as I get older.

Q30

##### Information

*Information* work describes an ongoing process of accumulating information to support tracking. This type of work is most analogous to Li et al’s [11] *preparation* stage, but we found that rather than engaging in just preparation, actors worked to accumulate a body of knowledge regarding aspects of tracking and illness. They used a wide variety of sources including other actors and third-party information sources, such as Wikipedia or medical websites, to learn terminology, make decisions, and understand feedback and outcomes (Q1). People with chronic illness and carers perform much of the information work, as it is specific to the individual’s experience of the illness.

In contrast to the preparation work described in Li et al’s model [11], information work informs people throughout the tracking process. Information can come from communication with other actors, such as health care providers:

I went and talked to my doctor about restless leg. We had a nice discussion about the genomic, the genetic aspects of this. He had some website stuff to go to.

Q9

One QS speaker describes doing information work while investigating patterns in her nutrition and symptom data. She engaged in this work simultaneously with reflection:

I got suspicious of bell pepper, tomatoes, and eggplant...It turns out they’re in the same family. It’s called nightshade. It has neurotoxins in it. They inhibit cholinesterase. What does cholinesterase do? Oh, my word. This...looks like what’s been happening.

Q11

As described by Valdez et al [8] in their patient work framework, *Information* and *Communication* work support the rest of the work of tracking.

##### Collection

*Collection* work involves data-gathering activities. Actors use tools (eg, glucometer, blood pressure cuff, journal, spreadsheet, and smartphone) to collect data (eg, numeric, text, or picture; objective or subjective) depending on the illness and health goals. Objective data include blood glucose levels, blood pressure, peak flow meter readings, geographic locations, and food intake. Subjective data include discomfort levels and degree of breathing difficulty.

Although most people recorded data in text or numeric form, some people used photos and video. These rich data types convey more information than a simple number and can be especially helpful in tracking food intake or changes in movement over time (Q20 and Q24). Speaker Q14 even used the quality of her handwriting in her headache journal to corroborate headache severity. Chung et al [25] similarly discuss types of data as well as boundary-negotiating artifacts generated through tracking.

Collection can also be collaborative, particularly in families [27]. For example, some parents of children with asthma share collection duties (eg, A6), especially if they share custody. Furthermore, spouses with similar conditions may track together (D6), and carers may track in collaboration with the people they support (Q25).

##### Integration

*Integration* work involves transforming data for analysis. People detailed the ways they collated and displayed data, with most people using a simple spreadsheet and graphs. Q10 describes how he visualizes the sneezes that are a symptom of his allergies:

This is a different way of looking at my sneezes. It’s a cumulative graph...and the slope indicated how fast I produce sneezes. So if it’s flat I don’t produce as many sneezes and if it’s very steep I produce a lot of sneezes in a short amount of time.

Q10

Integration work is usually performed to more deeply understand interactions between types of data, such as the effect of medication or treatment regimens on specific symptoms or the effect of stress on blood glucose levels. D4 showed his integration work for weight change and medication intake:

This is my chart that I made. I went into Excel...This is my weight. I weigh myself every day. See, I gained a couple pounds overnight...I’m going to have to...make sure I take three [medications] in the morning and three at night.

D4

Some people with chronic illness and carers use patient portals to make charts or tables with their data. A17 explained that she used her patient portal to integrate data:

I can chart my progress. I can see if my numbers are going up or going down, I can see my blood pressure. It’s not a test, but it’s on there and I can see what my blood pressure was when I went in for the visit.

A17

Many people drew inspiration to continue tracking from the visualizations they produced. Q17 described the information visualizations she used as “incredibly motivating.”

##### Reflection

*Reflection* work represents time spent engaging with data, making meaning from data, and considering the tracking experience itself. People with chronic illness or carers are usually primarily involved in reflection, with health care providers and community members providing additional insight. On the basis of an outcome, actors may decide to make adjustments or do something new. D8 reflected on how food intake affected her blood glucose:

I was writing down everything I ate during the...day and looking at the difference in my blood sugar, what caused it to be higher, and I had everything right there so that was more helpful.

D8

We also observed collaborative reflection work, consistent with previous research [14]. D1 discussed working with her pharmacist:

The pharmacist got involved in my cholesterol medication. She wanted me to go up a dose so we did a lot of communicating that way and that worked out.

D1

For an in-depth discussion of coordinated reflection, see Schroeder et al [32] discussing the work of people with Irritable Bowel Syndrome.

##### Action

*Action* work describes steps people take based on reflection or information work, often in collaboration with others. Some people talked about making incremental adjustments to their daily routine (Q19), but other speakers were inspired to make more substantial lifestyle changes, such as avoiding a medication (Tylenol for Q19) or cutting out foods (eliminating caffeine for Q3 to reduce panic attacks). Some also described weighing evidence from information and reflection work to synthesize conflicting advice from health care providers and decide on a plan of action (Q23).

A14 explained her new strategy for organizing medication after reflecting on gaps in medication logs:

I split them all up, and he was there [at his dad’s house] for two weeks so I bought several of these [pill organizers] because he takes one at night and three in the morning. So I put the three in here and the one at night and I just rubber banded these together. That’s how it’s foolproof. You don’t have to pack three different bottles and remember what combination.

A14

Community members can participate in action work, often providing support for improved illness management —such as taking medication consistently, keeping doctor’s appointments, or healthy eating. A12 explained how she kept her child’s school updated after changes in treatment plans:

I do a separate inhaler for school, I have a current prescription, I have Dr. A specifically sign on the paper saying this is the plan, this is how much she gets it if she needs it, she can or cannot carry it with her.

A12

People also update their tracking routine to sustain engagement. One person described his motivation for trying new tools and methods:

If you find a way to evolve the process frequently enough and meaningfully enough that you’re still excited about it as you go on, then I think that’s really powerful.

Q3

##### Dependencies and Workflow

Li et al [11] and Epstein et al [16] describe similar sequences of tracking work, with starting and ending points. Swan [42] describes types of work but no sequence, start, or end. We did not find a specific sequence but did find dependencies. We did not identify a definitive starting point but did identify common situations that trigger tracking. Owing to the ongoing nature of chronic illness, we did not observe a final ending to tracking, although 1 QS speaker did talk about tracking less frequently or discontinuing tracking during periods of better symptom control (Q14). Table 3 describes the dependencies among types of work. Table 4 describes 2 common situations that trigger tracking.

We found evidence for bidirectional transitions between each type of work, often involving handoffs between actors, and found that work does often overlap, corroborating Epstein et al [16] and Costa Figueirido et al [14]. For example, a parent may receive communication from a child’s teacher concerning a new symptom. Examining this exchange reveals a chain of rapid and interleaved tasks:

*collection* (teacher: symptom) → *integration* (teacher: past symptom data) → *reflection* (teacher: is this a new symptom?) → *communication* (teacher→parent: possible new symptom) → *integration* (parent: past symptom data) → *reflection* (parent: this is a new symptom) → *information/communication/ integration/ reflection* (parent: new symptom)

**Table 3:** Dependencies among types of work.

Dependency	Description
(Collection, Information) → Reflection	Reflection work cannot take place without some kind of collection or information work (eg, a weight measurement or list of medication side effects)
Collection → Integration	Integration work cannot take place without at least 1 data point each of 2 types of data (eg, a meal photo with a blood glucose measurement)
(Information, Communication, Reflection) → Action	As we defined it, action work is a change of plan for managing the illness and must be based on the outcome of other work, usually information, communication, or reflection work

**Table 4:** Common triggers for tracking.

Trigger	Description
Collection	Collection (ie, observed symptoms or abnormal test result) leading to reflection (ie, “what does that high blood pressure result mean?”)
Information or Communication	Incidental information or communication (ie, reading a magazine article or talking to a friend) gives rise to reflection (ie, “is that why I’ve been feeling tired?”) and collection (ie, “let’s investigate”)

**Figure 1:**
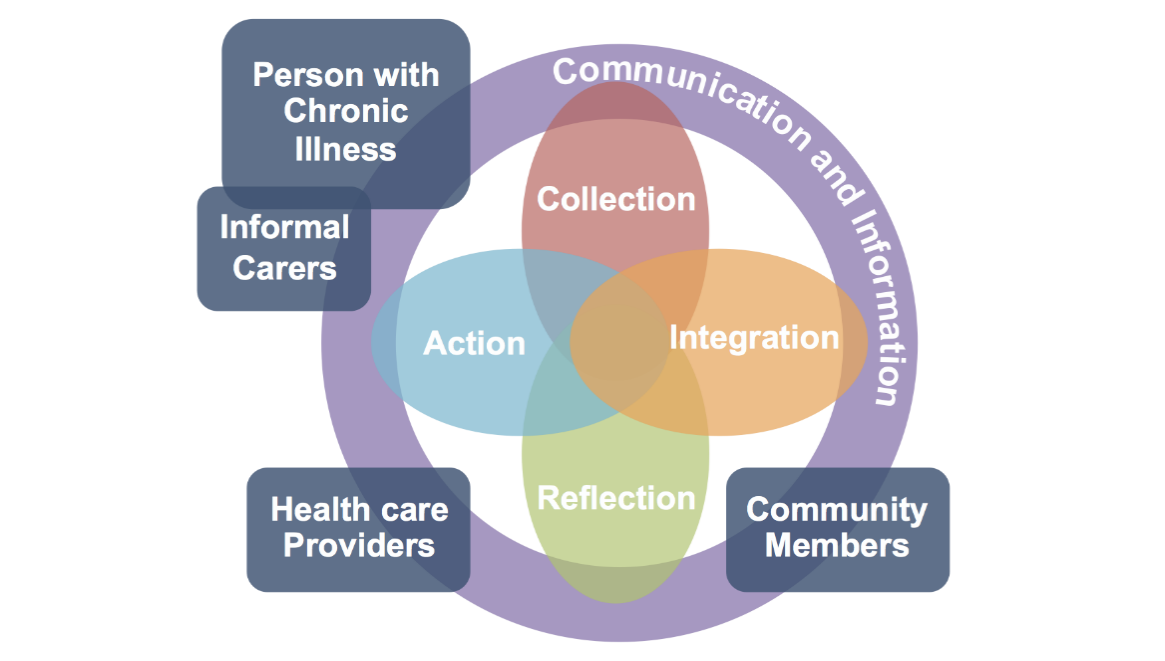
The Conceptual Model of Shared Health Informatics (CoMSHI) showing the work and social context of tracking in support of chronic illness management and the interplay between components. Actors are the person with chronic illness, informal carers, community members, and health care providers. The work in which those actors engage includes communication, information, collection, integration, reflection, and action. Work is done in no particular order, and types of work can overlap. All actors may engage in work and interact with each other around that work.

The parent’s work in the last step combines information work interleaved with integration, reflection, and communications work concerning how the new symptom and information fit with her previous understanding of information and data (eg, reflection-in-action [54]).

We developed a conceptual model of the tracking process that supports chronic illness management based on what we learned.

#### Conceptual Model of Shared Health Informatics

To bridge the gap between current informatics models and important characteristics of tracking for chronic illness management, we proposed the CoMSHI (pronounced *com-she*; Figure 1). The CoMSHI is based on insights from previous research (eg, [11,14,20,21,24,42,47]) and new data analysis about tracking behavior and social interactions. It portrays the actors and work, described above, that drive successful tracking in support of chronic illness management. Actors perform work in no particular order, and work can be ongoing and overlapping. All actors may engage in work and communicate around that work.

## Discussion

In this study, we aimed to construct a model in response to informatics literature indicating a need for better representation of the unique challenges and context around tracking to support illness management [13,14,47]. We combined insights from the literature with an analysis of 69 interview and video transcripts to develop the CoMSHI.

### Contributions

The CoMSHI extends previous work on personal and health informatics models; Table 5 summarizes a comparison with that previous work. Our model is unique in describing the relationships among people and work involved in tracking in support of chronic illness management and emphasizes communication and shared work.

Table 6 summarizes the contributions of this study. A valuable extension to previous personal and health informatics models is the inclusion of *carers*. Although Pew’s health tracking survey [6] found that 12% of trackers track for someone else, no other informatics model includes carers as primary actors and trackers. To address this gap observed in the literature and our data analysis, we have highlighted that carers often assume a critical role in tracking to manage chronic illness. We modified Li et al’s stages of work to unconstrained transitions between work because people managing chronic illness do not progress through a sequence of stages but continuously and iteratively work in support of their health [7]. We redefined the *preparation* stage to *information* work. This reflects the ongoing knowledge building that supports the other work and actors. Our analysis also showed that shared work among the person with chronic illness, carer, community members, and health care providers was key to successful tracking, in line with findings of other research outlined in the related literature.

**Table 5:** Characteristics of the Conceptual Model of Shared Health Informatics (CoMSHI) compared with models from studies by Li et al, Epstein et al, Swan, and Murnane et al.

Model	Model description and basis	Role of tracker	Work	Roles of others	Outcomes
Stage-Based Model of Personal Informatics [11]	Literature analysis, empirical study defining personal informatics	One person who performs all work	Preparation, collection, integration, reflection, action	N/A^a^	Increased self-knowledge, informed action
Lived Informatics Model of Personal Informatics [16]	Literature analysis, empirical study defining lived informatics	One person who performs all work	Deciding, Selecting, tracking and acting, lapsing	N/A	Increased self-knowledge, informed action, lapsed tool use with possible resumption
Patient-Driven Health Care Model [42]	Description of patient-driven health care	One patient who performs all work	Research, treat, intervene, experiment, track, measure	Patient initiates contact with peers and professionals	Self-expression, enhancement, prevention, cure, normalization, improvement
Model of the Sociotechnical Ecology Surrounding Serious Mental Illness Management [43]	Literature analysis, empirical study defining social relations in managing severe mental illness	One patient who performs work and is influenced by external actors and contexts	N/A	Patient interacts with close ties, institutions, sociocultural context	Interpersonal comparisons and baselines, mitigation and management of crises
Conceptual Model of Shared Health Informatics	Literature analysis, empirical study defining people and work in tracking to manage chronic illness	One or more people who communicate and share tracking work	Communication, information, collection, integration, reflection, action	Part of the social ecology communicating and supporting tracking work	Increased knowledge, communication**,** informed action

^a^N/A: not applicable.

**Table 6:** Contributions of the Conceptual Model of Shared Health Informatics (CoMSHI).

Contribution	Description
Carer as primary actor	Carers often assume a critical role in tracking to manage chronic illness
Communication work	Communication work supports interactions among actors around tracking work
Information and communication work support tracking practice	Information and communication work are the backbone enabling exchange of ideas and insights as well as transitions between work
Distributed work	Tracking work is distributed across multiple actors rather than resting only with one person
No prescribed work sequence	Work is ongoing, nonsequential, and sometimes overlapping rather than linear and time-limited

### Implications for Design

As are previous models, the CoMSHI is agnostic to specific tools used or data elements collected. Rather, the model describes the relationships among work and people that health informatics tools need to accommodate. New health informatics tools would better align with the experience of people involved in tracking for chronic illness management if designed to support both the types of work and actors involved, thus promoting effective management and potentially improving health outcomes. On the basis of the CoMSHI, we recommend that, early in the design process, designers determine the extent of the tracking practice their tool will support and then define the functionality necessary to facilitate shared work and transitions between people and types of work. Any one tool does not need to support all aspects of tracking work, but designers must critically consider how to empower people to track the data they need, collaborate with whom they choose, and transition between tools that support other tracking tasks. The first step in accomplishing this goal is to develop a deep understanding of the users of the technology, their goals for tracking, and their illness or illnesses. Without thoughtful engagement with people and work, designers will find it difficult to create truly usable and useful technology for those they serve.

### Limitations and Future Work

This model is based upon transcript analysis of people managing chronic illnesses and as such we can only claim that it applies to that context. However, based on our understanding of the literature, it may apply to tracking in other health contexts such as for people who are hospitalized [47] or people with cancer [52,55]. We also did not interview representative health care providers or community members for their perspectives. Further research is needed to evaluate generalizability. Also, several articles [28,29,56] assert that the privacy policies around health information technology, especially patient portals, are insufficient to effectively support the needs of people with chronic illness and carers. Our study suggests that further research should also consider the role of community members to ascertain how to best support the work and social ecosystem of tracking in support of chronic illness.

### Conclusions

For people managing chronic illness, effective tracking improves health outcomes. Health informatics tools intend to help but they often fall short of supporting the true range of work and people involved. Furthermore, current research and tools often focus on *personal* informatics, *self*-management, or *self*-tracking—limiting how we think about and design to support tracking for chronic illness management. Understanding the shared work of tracking can inform the design of systems to support the reality of managing chronic illness [7,8]. One QS speaker asserts: “in chronic diseases, health is not created in healthcare (Q16),” emphasizing that she cannot rely solely on health care providers and her tracking practice supports her health in the life outside the clinic.

This study has contributed a model of the work and social context of tracking in support of chronic illness management to advance the understanding of how to support successful health tracking. The CoMSHI gives insight into the processes used by people who successfully manage a chronic illness as well as the context in which they work. The CoMSHI expands on its predecessors by (1) including informal carers, (2) emphasizing the shared nature of tracking work, and (3) characterizing work as ongoing and nonsequential. This new model demonstrates the fluidity of the tracking process and situates the work of tracking in its social context. Most importantly, this work underscores the impossibility of isolating tracking work from the social environment of people managing chronic illness, and designers must consider the shared aspects of tracking when designing health informatics tools. Although previous models focus on a single person engaging in tracking work, the CoMSHI emphasizes that it is only part of the puzzle. As one speaker expressed, “It’s not just technology, it’s people” (Q21).

## Abbreviations

AHRQ: Agency for Healthcare Research and Quality
CoMSHI: Conceptual Model of Shared Health Informatics
HIPAA: Health Insurance Portability and Accountability Act
NIH: National Institutes of Health
QS: Quantified Self
